# The Clinical Impact of a Multidisciplinary Strategy: Diagnostic and Therapeutic Flow-Chart for Vertebral Metastases with Spinal Cord Compression

**DOI:** 10.3390/cancers17091479

**Published:** 2025-04-28

**Authors:** Rossella Rispoli, Fabrizia Giorgiutti, Claudio Veltri, Edi Copetti, Pietro Imbrucè, Giorgia Iacopino, Barbara Cappelletto

**Affiliations:** Ospedale Universitario Santa Maria Della Misericordia di Udine, 33100 Udine, Italy; fabrizia.giorgiutti@asufc.sanita.fvg.it (F.G.); claudo.veltri@asufc.sanita.fvg.it (C.V.); edi.copetti@asufc.sanita.fvg.it (E.C.); barbara.cappelletto@asufc.sanita.fvg.it (B.C.)

**Keywords:** spinal cord, metastatic spinal cord compression, multidisciplinary, neurological deterioration

## Abstract

**Introduction:** Metastatic spinal cord compression (MSCC) is a life-threatening complication caused by the involvement of the spinal cord or nerve roots. It can result in severe neurological deficits, including paralysis, sensory loss, and bladder or bowel dysfunction, significantly affecting patients’ quality of life. Prompt diagnosis and intervention are critical to minimizing these outcomes and improving neurological recovery. This study evaluates the efficacy of a diagnostic and therapeutic protocol introduced at our institution in 2022, designed to enhance early detection, optimize management, and improve outcomes for patients with MSCC. **Materials and Methods:** The protocol was developed through a multidisciplinary collaboration involving emergency physicians, oncologists, spine surgeons, neuroradiologists, and radiation oncologists. Each specialty contributed its expertise to create a streamlined approach emphasizing early symptom recognition, rapid diagnostic imaging, and timely therapeutic interventions, such as surgical decompression and radiotherapy. To assess the protocol’s effectiveness, a retrospective analysis was conducted. Clinical data from the pre-implementation period (years 2019–2021) were compared to the post-implementation period (2022–2024). **Results:** The implementation of the algorithm significantly improved MSCC management, increasing outpatient consultations from 671 to 828 (+23%). Comparing the pre- to post-implementation periods, emergency consultations rose from 14% to 23%, while intrahospital consultations decreased from 20% to 16%. Surgical procedures increased slightly, from 60 to 66 (+10%), including 26 emergency surgeries (+4%) and 40 elective delayed surgeries (+14%). **Conclusions:** The introduction of a multidisciplinary diagnostic and therapeutic protocol significantly improved outpatient management of patients with metastatic spine disease, demonstrated by a significant increase in urgent outpatient consultations and a reduction in intrahospital consultations. The number of surgical interventions for metastatic spinal cord compression slightly increased after the protocol’s adoption, although the proportion of elective surgeries remained unchanged. Neurological presentation at the time of surgery did not show a statistically significant difference between the pre- and post-implementation periods. These findings highlight the protocol’s effectiveness in optimizing patient flow and triaging, while further research is needed to evaluate its long-term clinical impact.

## Simple Summary

Metastatic spinal cord compression (MSCC) is a serious condition that can lead to neurological deficits significantly affecting patients’ quality of life. Early diagnosis and treatment are essential to improving patient outcomes. This research evaluates a new algorithm introduced in 2022 at our institution, designed to speed up diagnosis and optimize treatment. A team of specialists, including emergency doctors, oncologists, surgeons, and radiologists, collaborated to create a structured approach focusing on early symptom recognition and rapid intervention. Comparing patient data from before and after the protocol was implemented, we observed an improvement in consultations and surgeries, leading to better patient care. These findings suggest that a well-organized, multidisciplinary approach can enhance MSCC management and reduce neurological decline. In the future, advancements in artificial intelligence could further improve diagnostic accuracy, risk prediction, and treatment timing, enhancing the overall effectiveness of MSCC management strategies.

## 1. Introduction

Spinal metastases are a frequent and significant complication of advanced cancer, arising in up to 60% of patients with metastatic disease due to the vertebral column’s extensive vascular and lymphatic networks. Advances in chemotherapy, immunotherapy, and radiation therapy have extended survival rates but have also led to an increased prevalence of spinal metastases, now reported in approximately 20% of cancer patients [1,2]. Among these cases, 25–50% develop symptomatic metastatic spinal cord compression (MSCC), a condition characterized by high morbidity, functional impairment, and reduced quality of life [3,4].

In some cases—up to 20%—MSCC is the initial clinical manifestation of cancer, particularly in aggressive malignancies such as lung cancer, multiple myeloma, and non-Hodgkin lymphoma. Accurate histological diagnosis in such cases is critical for guiding treatment strategies. The thoracic spine is most commonly affected, and lesions in this region frequently lead to severe neurological deficits, including paralysis, sensory impairments, and sphincter dysfunction. These complications significantly impact patient independence, quality of life, and survival, placing a heavy burden on patients and caregivers alike [5,6].

The management of MSCC requires a multidisciplinary approach involving emergency physicians, oncologists, spine surgeons, neuroradiologists, and radiation oncologists. Early diagnosis is crucial, limiting treatment options and worsening prognosis [7].

This study evaluates the effectiveness of a diagnostic and therapeutic algorithm designed to optimize the care of patients with spinal metastases and MSCC. The algorithm focuses on early symptom recognition, seamless interdisciplinary collaboration, and prompt therapeutic interventions to prevent irreversible neurological damage [8]. By addressing these critical elements, the approach aims to improve patient outcomes, preserve functional independence, and enhance quality of life for individuals facing the challenges of MSCC.

## 2. Materials and Methods

A multidisciplinary protocol for managing MSCC was established at a primary care center. This protocol involves emergency physicians, spine surgeons, neuroradiologists, radiation oncologists, and oncologists, with each specialist assigned a specific role in the care process [9].

The protocol applies to all the individuals presenting to the emergency department with symptoms of root or spinal cord compression, regardless of a previous oncological history. The anamnestic data of a previous oncological history may help guide the diagnosis, but it does not influence the application of the protocol. In cases of previously undiagnosed cancer, the radiation oncologist is alerted once the MRI is done.

The approach begins with the identification of MSCC symptoms—such as nocturnal or mechanical axial pain, sudden spinal, or radicular pain, gait instability, limb weakness, or bladder and bowel dysfunction—by emergency physicians or oncologists (Table 1). These professionals promptly engage spine surgeons and radiation oncologists. Spine surgeons perform detailed neurological evaluations, arrange for magnetic resonance imaging (MRI) with neuroradiologists, and devise treatment plans. When there is a non-univocal interpretation of the MRI findings, a multidisciplinary discussion involving neuroradiologists, oncologists, and spine surgeons is conducted to reach a consensus.

We apply the NOMS (neurological, oncological, mechanical, and systemic) framework to guide the decision-making process, ensuring a structured and consistent approach to patient management.

Neurological status at first presentation was assessed using the American Spinal Injury Association (ASIA) Impairment Scale. For the purpose of analysis, patients were grouped into two categories: those with severe neurological deficits (ASIA grade A, B, or C) and those with mild or no deficits (ASIA grade D or E).

In cases involving severe compression or mechanical instability, surgical decompression and stabilization are prioritized, often performing a “separation surgery” to prepare the field for advanced radiotherapy techniques. Surgical procedures are performed urgently for patients with severe neurological deficits or rapidly progressive deficits. In this study, urgent surgical intervention was defined as surgery performed within 72 h from the initial evaluation by the spine surgery team. Indications for urgency included clinical and/or radiological evidence of neurological deterioration and/or mechanical instability. Patients with acute severe neurological deficits occurring more than 48 h before admission were not classified as urgent cases if their condition had stabilized by the time of evaluation. Surgery is deferred for patients without neurological deficits or with mild, stable deficits. The delay is primarily intended to ensure proper patient staging, optimize surgical planning by performing angiography to assess spinal cord afferences and potential embolization, or address significant comorbidities and preoperative optimization needs, such as anticoagulation management.

Radiation oncologists focus on treating vertebral metastatic tumors to achieve local tumor control and, in some cases, preserve neurological function. Oncologists, physiatrists, and palliative care specialists provide systemic therapy, rehabilitation, pain control, and emotional support to ensure comprehensive patient care.

**Figure 1:**
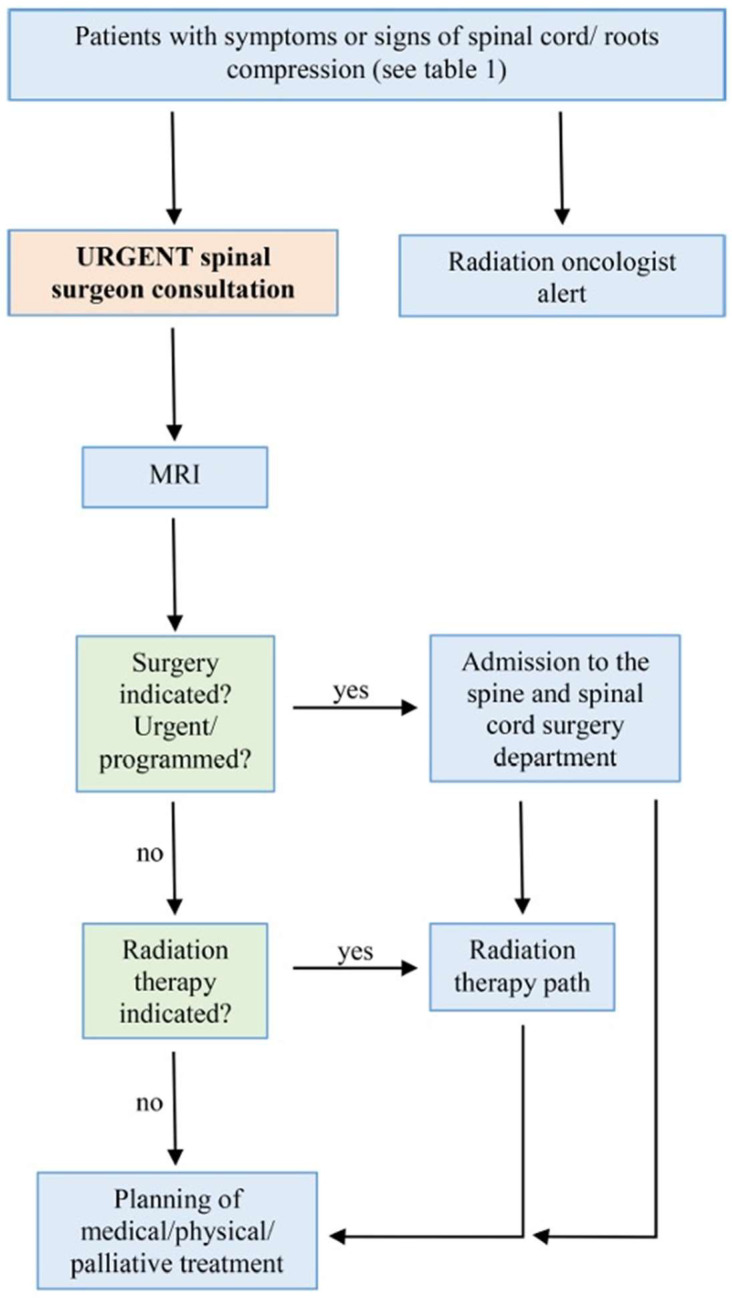
The algorithm we introduced and began applying in 2022.

The algorithm we introduced and began applying in 2022 (9) is depicted in detail in Figure 1, offering a visual representation of its structure.

To evaluate the impact of the algorithm, we compared clinical outcomes from 2019–2021 (pre-implementation) with those from 2022–2024 (post-implementation). The analysis included all the patients diagnosed with vertebral metastases assessed by the spine surgeons in our unit. Data were collected on patients seen in emergency settings as well as those evaluated through intrahospital consultations. Additionally, we analyzed the number and type of surgical procedures performed, differentiating between emergency and elective surgeries. We used binomial and chi-square tests (Microsoft Corporation, 2024, Redmond, WA, USA), as appropriate based on the size of the sample, to compare proportions before and after algorithm implementation.

## 3. Results

**Figure 2:**
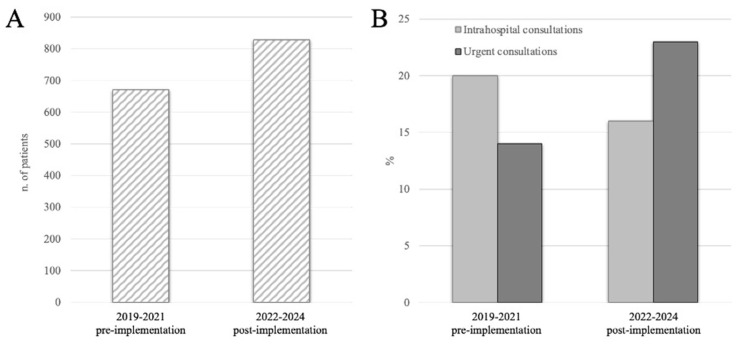
(**A**) Total number of patients evaluated by spine surgeons in our unit, revealing a 23% increase. (**B**) Percentage of these patients seen as intrahospital consultations (light grey bars), and as urgent consultations (dark grey bars). Comparing the pre-implementation to the post-implementation periods, intrahospital consultations decreased, and urgent consultations increased.

The total number of oncological patient evaluations raised in the period post-implementation compared to the pre-implementation period. Between 2022 and 2024, 828 patients with metastatic spine disease were assessed, marking a 23% increase compared to the 671 patients evaluated between 2019 and 2021 (Figure 2A). This growth reflects the protocol’s impact on raising awareness and expanding access to care.

The implementation of the algorithm led to considerable changes in consultation dynamics. Intrahospital consultations decreased from 20% in the pre-implementation period to 16% post-implementation (135 out of 671 vs. 132 out of 828). This decrease was statistically significant (binomial test, *p* = 0.037), suggesting improved triaging efficiency and better coordination between departments. Meanwhile, outpatient urgent visits increased significantly (94 out of 671 vs. 190 out of 828), rising from 14% to 23% (Figure 2B). This increase was statistically significant (binomial test, *p* < 0.001), reflecting a shift towards more proactive, targeted, and continuous management of patients with spinal metastases in outpatient settings.

**Figure 3:**
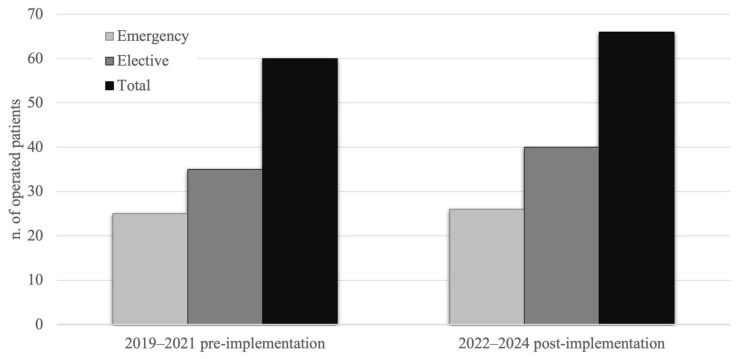
The graph illustrates the surgical modalities of patients operated on in our unit across two periods. The light grey bars represent emergency procedures, which increased by 4%, while the dark grey bars represent elective surgeries, which increased by 14%, comparing the pre-implementation and post-implementation periods. The black bars display the total number of surgeries performed, showing an overall 10% growth.

The number of surgical interventions for spinal cord compression increased following the adoption of the protocol from 60 to 66 (+10%). During the pre-implementation period, 60 surgeries were performed, comprising 25 emergency and 35 elective delayed procedures. Post-implementation, the total increased to 66 (+10%), with 26 emergency (+4%) and 40 elective delayed surgeries (+14%). These results are illustrated in Figure 3.

The proportion of elective interventions to all interventions was unchanged after adoption of the protocol (42% vs. 39%, Chi-square test, *p* = 0.938). 

**Figure 4:**
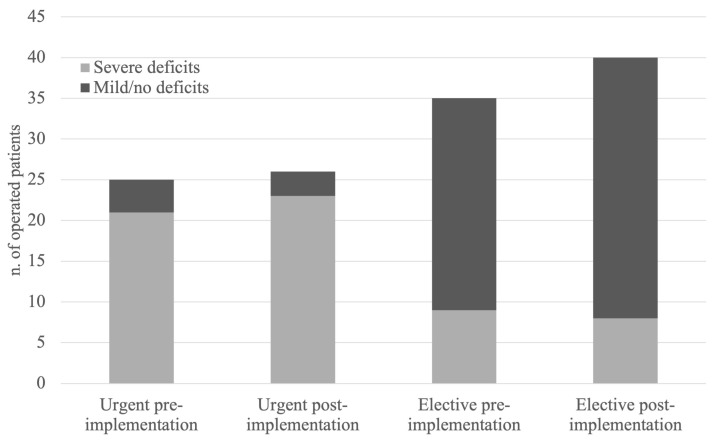
The graph illustrates the comparison of neurological deficits in urgent versus elective surgeries, before and after the protocol implementation. The light grey portion of each bar represents patients with severe neurological deficits, accounting for 84% and 88% of urgent cases, and 26% and 20% of elective cases, in the pre- and post-protocol periods respectively. The dark grey portion represents patients with mild or no neurological deficits.

Among the 25 patients who underwent urgent surgery during the pre-protocol period, 21 (84%) presented with severe neurological deficits, while 4 (16%) had mild or no deficits. Among the 35 patients who underwent elective surgery during the same period, 9 (26%) exhibited severe neurological impairments, and 26 (74%) had mild or no deficits. Following the implementation of the protocol, among 26 patients who underwent urgent surgery, 23 (88%) experienced severe neurological deficits, and 3 (12%) had mild or no deficits. Meanwhile, of the 40 patients who underwent elective surgeries, 8 (20%) had severe deficits, whereas 32 (80%) presented with mild or no neurological impairment. The distribution of neurological outcomes (severe vs. mild or no deficits) before and after protocol implementation showed no statistically significant difference for either urgent or elective surgeries (binomial test: *p* = 0.524 for urgent surgery; *p* = 0.850 for elective surgery). These data are illustrated in Figure 4.

## 4. Discussion

The management of spinal metastases is inherently complex and necessitates a multidisciplinary approach. Each patient presents unique challenges based on a variety of factors, including the type of primary cancer, extent of metastatic spread, general health, and response to initial treatments. Therefore, no single treatment approach fits all cases; instead, individualized treatment plans must be crafted, incorporating a wide range of medical specialties [10,11]. The involvement of various professionals from different fields enables a holistic treatment strategy that maximizes the potential for improved patient outcomes [12].

Central to this multidisciplinary care is the integration of specialized algorithms and scoring systems, which provide a systematic approach to decision-making, ensuring that all clinical parameters are considered and addressed. The Spine Oncology Consortium (SOC) framework, widely respected and utilized, provides an evidence-based structure for determining appropriate therapeutic interventions, including radiotherapy, surgical interventions, and neuro-interventional techniques. These treatments may be utilized independently or in combination, depending on the specific needs of the patient, the characteristics of the metastatic lesions, and the patient’s overall health status [13].

Two essential clinical algorithms—NOMS (neurological, oncological, mechanical, and systemic) and LMNOP (local disease, metastatic disease, neurological status, overall prognosis, and pain)—are used to assess the critical parameters that guide clinical decision-making [14,15]. These algorithms allow for a more personalized treatment plan by evaluating a broad range of factors, including the neurological status of the patient, oncological diagnosis, the mechanical integrity of the spine, the systemic health of the patient, and the pain associated with the metastatic disease. This systematic approach enhances clinical precision and reduces subjectivity, helping to ensure that patients receive the most appropriate and effective treatment for their specific situation.

Another pivotal tool, the Spinal Instability Neoplastic Score (SINS), allows for the quantification of spinal instability due to metastatic lesions. It assesses the location of lesions, pain intensity, and the structural integrity of the spine. These variables are evaluated using a scoring system that helps clinicians determine whether surgical intervention is necessary and the type of surgery that might be most appropriate [16,17]. This scoring system helps clinicians avoid unnecessary procedures and ensures that surgical interventions are targeted to patients who will benefit the most, ultimately reducing risks and improving clinical outcomes.

### 4.1. The Role of Early Detection and Diagnosis

The timely identification of spinal metastases is critical in preventing MSCC, a life-threatening condition that can result in irreversible neurological damage. Early detection of MSCC significantly improves the chances of successful treatment and better patient outcomes. The earlier MSCC is diagnosed, the more treatment options become available, thus increasing the possibility of saving neurological function [18,19].

Whole-spine magnetic resonance imaging (MRI) remains the gold standard for diagnosing spinal metastases and assessing the presence of spinal cord compression. Whole-spine MRI offers high-resolution imaging, enabling the detection of even small metastatic lesions that may otherwise be overlooked. Moreover, it allows clinicians to visualize the full extent of the metastases, ensuring that treatment decisions are made based on the most accurate and comprehensive information. However, computed tomography (CT) is frequently employed as a complementary tool, especially in the context of surgical planning, where the precise delineation of anatomy (such as pedicle size) and the characteristics of bone metastases (lytic or blastic) is essential. In patients with pacemakers or other contraindications, CT serves as a screening tool.

To further streamline the diagnostic process and minimize delays in treatment, innovative solutions such as telephone-based symptom screening and accelerated access to MRI have proven to be highly effective [20,21]. These strategies aim to reduce the time between symptom onset and diagnosis, ensuring that patients receive prompt care. The importance of this approach has been reinforced by international guidelines, such as those from the National Institute for Health and Care Excellence (NICE), which support the use of these systems to facilitate early diagnosis, thereby preventing unnecessary delays in treatment initiation. Reducing diagnostic delays is particularly important in high-risk populations where the progression of spinal metastases can occur rapidly, making early intervention a critical factor for achieving favorable outcomes [22,23].

### 4.2. A Multidisciplinary Protocol for Spinal Metastases Management

A multidisciplinary protocol is essential for the comprehensive management of spinal metastases, ensuring that care is not only effective but also patient-centered. This protocol is designed to streamline the care pathway, reducing delays between diagnosis and treatment and facilitating timely interventions that help prevent permanent neurological damage. With an emphasis on early detection and immediate action, the protocol integrates the efforts of multiple specialists across various stages of care [9].

The multidisciplinary team consists of experts from numerous fields, including spine surgeons, neuroradiologists, radiation oncologists, medical oncologists, physiatrists, and palliative care specialists. These professionals collaborate closely to develop individualized care plans, ensuring that all aspects of a patient’s condition are considered. This collaborative approach ensures that no aspect of care is overlooked, from the initial diagnostic imaging to the final stages of treatment and rehabilitation.

Involving patients in the decision-making process is fundamental. By actively engaging patients in their treatment plans and ensuring that their preferences and values are respected, the protocol fosters a shared decision-making environment. This patient-centered approach has been shown to enhance patient satisfaction and can significantly improve outcomes, as it ensures that the care plan aligns with what is most important to the patient [24].

Early full-spine MRI imaging is a central component of the protocol, enabling clinicians to quickly assess the extent of metastatic spread across the entire spine. This allows clinicians to prioritize the most urgent cases and initiate appropriate interventions promptly. Our primary objective remains the preservation or recovery of neurological function. However, when the metastatic origin and spread are unclear and may significantly influence treatment decisions, additional imaging, such as a PET-CT scan, may be performed. While ensuring timely neurological intervention, a deferred surgical approach may be considered, allowing for a more comprehensive diagnostic evaluation and optimized treatment planning.

For patients with spinal instability, localized disease, or good functional status, surgery is often recommended as the treatment of choice, aiming to alleviate pain, stabilize the spine, and improve functional outcomes. For patients with more widespread disease or those with a limited prognosis, radiation therapy may be more appropriate.

### 4.3. Impact of the Protocol: Transforming Clinical Outcomes and Surgical Management Improvements

The implementation of the multidisciplinary protocol has yielded substantial improvements in patient care, as evidenced by the number of patients evaluated between 2022 and 2024. A total of 828 patients were assessed during this period, representing a considerable increase (23%) compared to the 671 patients evaluated between 2019 and 2021. The 2019–2021 timeframe was affected by the COVID-19 pandemic, which disrupted hospital admissions and referrals, leading to a temporary reduction in reported cases. The post-implementation period (2022–2024), in contrast, reflects a return to regular patient flow and an increase in consultations, particularly emergency consultations, which rose from 14% to 23%. This suggests that the protocol contributed to improved patient identification and triaging, rather than simply reflecting an arbitrary change in volume. Moreover, this rise in patient evaluations demonstrates the growing recognition of the protocol’s effectiveness in enhancing care for this often-neglected group of patients with metastatic spine disease.

Although the proportion of elective interventions relative to all interventions did not change significantly after adoption of the protocol, the growing recognition of the algorithm’s value in the early diagnosis and management of metastatic spine disease likely contributed to more timely and better-targeted interventions. While the percentage of surgical interventions relative to total cases (7.9% post-implementation vs. 8.9% pre-implementation) slightly decreased, the absolute number of surgical interventions increased by 10% (from 60 to 66 cases). This rise in surgical interventions directly reflects the protocol’s emphasis on early detection and comprehensive management, allowing for the prompt identification of patients who are most in need of surgery. Notably, elective surgeries saw a 14% increase when comparing the pre- and post-implementation periods. This increase reflects a shift towards better-planned, proactive interventions rather than reactive emergency surgeries. The protocol’s goal was not to increase surgeries but to optimize patient selection and treatment timing, ensuring that patients receive care at the optimal stage of disease, before neurological symptoms of spinal cord compression emerge. In the post-protocol period, 88% of patients undergoing urgent surgery presented with severe neurological deficits, compared to 84% in the pre-protocol period. Conversely, only 20% of patients undergoing elective surgery in the post-protocol period had severe deficits, compared to 26% previously. Although the distribution of neurological outcomes (severe vs. mild or no deficits) before and after protocol implementation did not show a statistically significant difference for either urgent or elective surgeries, this trend indicates improved alignment between neurological severity and surgical timing. These findings suggest that the algorithm effectively prioritized urgent cases and appropriately deferred elective interventions for patients with mild or no deficits.

The 2022–2024 years patient evaluation rise not only highlights the growing effectiveness of the protocol but also indicates a broader transformation in how spinal metastases are being identified and treated. By shifting the focus to earlier intervention, the protocol has enabled more patients to receive timely treatment when therapeutic options are broader, and outcomes are more favorable.

The protocol’s success is also reflected in the shift toward outpatient care. Over the course of the protocol’s implementation, intrahospital consultations slightly decreased from 20% to 16%, while outpatient visits saw a substantial increase, rising from 14% to 23%. This trend reflects an improvement in consultation dynamics, such as a decrease in intra-hospital consultations, indicating fewer inpatients in severe condition, and an increase in urgent outpatient visits, suggesting more stable patients managed outside the hospital. This suggests that the protocol enhanced coordination across departments, leading to earlier and more appropriate interventions, while also empowering patients to take a more proactive role in their care. Outpatient care is often less costly and more flexible, allowing patients to manage their condition with less disruption to their daily lives.

## 5. Limitations

This study has several important limitations that need to be addressed for a more accurate interpretation of its findings. First, the data from 2020 and 2021 may be influenced by the COVID-19 pandemic, potentially distorting trends or changes during that time, and impacting the study’s conclusions. Second, the single-center design limits the ability to generalize the findings to other settings or populations, reducing the external validity of the results. To enhance the robustness of the findings, long-term studies are essential to capture broader trends and fluctuations over time, providing a more comprehensive understanding. Moreover, multicenter studies would offer a more diverse sample, allowing for validation across different populations and a deeper exploration of underlying factors. Third, our primary aim was to evaluate improvements in patient access and care coordination for individuals with spinal cord compression. However, critical indicators of treatment success and long-term patient outcomes—such as functional recovery, and overall survival—were not considered in this analysis. These factors will be incorporated into future studies to provide a more comprehensive assessment of the protocol’s impact.

Addressing these limitations through broader research would improve the reliability and applicability of the findings.

## 6. Future Directions

The integration of artificial intelligence (AI) into medical diagnosis represents a groundbreaking shift with the potential to revolutionize healthcare across multiple dimensions. One of its most powerful advantages is its ability to efficiently process and analyze vast amounts of medical data at a scale and speed far beyond human capabilities. This computational power not only accelerates the diagnostic process but also enhances its accuracy, reducing the likelihood of human error and biases, which often influence clinical decisions. The result is more precise diagnoses, faster decision-making, and more reliable outcomes for patients. In the context of vertebral metastases, AI has the potential to significantly improve early detection and intervention, ensuring that patients are recognized before the neurological signs of spinal cord or root compression manifest. Our algorithm, which we have implemented to improve the recognition of patients with spinal metastases, could take advantage of AI’s analytical power to identify individuals at heightened risk and prioritize them for timely interventions. By leveraging patient data such as imaging results, medical history, and cancer type, the algorithm, powered by AI, could enhance the decision-making process regarding the optimal timing and type of surgical intervention, preventing or minimizing neurological compromise.

In addition to improving diagnostic accuracy, AI offers significant potential for disease prediction and prevention. By harnessing a wide array of patient data—including medical history, lifestyle choices, environmental factors, and genetic information—AI algorithms can identify individuals at heightened risk for various conditions [25]. This predictive capability allows for early intervention strategies, such as targeted screenings or preventive measures, to be implemented before the disease reaches an advanced stage. In the case of MSCC, this means detecting spinal metastases earlier in their progression, before they result in irreversible neurological damage. Our algorithm could play a key role in this by integrating real-time data analysis to flag at-risk patients and ensure that appropriate care is administered without delay. Early detection of spinal metastases through AI-powered systems could enable clinicians to initiate treatments, such as surgery or radiation, at the right time.

By analyzing a patient’s medical records, current health status, comorbidities, and responses to previous treatments, AI can generate personalized recommendations tailored to the specific needs of the individual. This not only ensures that patients with spinal metastases receive the most appropriate care based on their unique circumstances but also enhances the efficiency of healthcare delivery by supporting clinicians in making the most informed decisions possible. In particular, our algorithm could help prioritize patients with vertebral metastases who are at risk of MSCC, ensuring they receive timely surgical treatment before the onset of neurological symptoms. Furthermore, AI’s ability to analyze large datasets and identify trends can assist in resource allocation, ensuring that healthcare providers can distribute limited resources more effectively, particularly in high-demand settings. In the case of spinal metastases, this means ensuring that patients who need urgent care are seen promptly, preventing delays that could result in irreversible spinal damage.

Ultimately, the application of AI in healthcare holds immense potential to transform the way we approach medical diagnosis, disease prevention, and treatment. By streamlining the diagnostic process, enhancing early detection and intervention, and providing personalized treatment plans, AI is poised to improve both the quality of care and the overall efficiency of healthcare systems. In the context of spinal metastases and MSCC, the integration of AI—through our algorithm—could dramatically improve patient outcomes by enabling earlier detection, optimizing the timing of surgical interventions, and minimizing neurological damage. As AI technology continues to evolve and integrate more deeply into clinical practice, its capacity to reshape the healthcare landscape will continue to expand, offering valuable opportunities to improve patient care. The ongoing development of AI-powered tools in medicine is not just a technological evolution—it is a paradigm shift in how healthcare is delivered and experienced, offering promising solutions to some of the most pressing challenges facing modern medicine.

## 7. Conclusions

Building on its proven success, the protocol is being strategically extended to include peripheral hospitals and primary care settings. This initiative integrates general practitioners and smaller healthcare facilities, enabling patients to access timely interventions within their local communities. By reducing the need for referrals to centralized, often distant, specialized centers, this approach addresses critical delays in care, ensuring that patients receive prompt and effective treatment.

This expansion is expected to significantly enhance survival rates, functional recovery, and the overall quality of life for patients with MSCC [26,27]. It not only ensures that patients benefit from faster, more convenient care but also optimizes healthcare resource utilization. Decentralizing services helps to alleviate the growing pressure on larger, centralized facilities, fostering a more balanced and sustainable healthcare system.

The comprehensive approach focuses on maintaining consistency in care delivery while prioritizing patient-centric outcomes. By empowering local practitioners with the tools, training, and resources needed to manage MSCC effectively, the protocol ensures a seamless continuum of care. This reduces the physical and emotional burden on patients and their families, minimizes treatment delays, and enhances collaboration across all levels of the healthcare system. Ultimately, this model promotes a more resilient and equitable system that benefits both patients and providers.

Drawing on the advancements in AI, these efforts could potentially leverage AI’s analytical power to improve diagnostic accuracy, predict risks, and optimize treatment timing, thereby enhancing the overall effectiveness of the algorithm in managing MSCC.

## Figures and Tables

**Table 1:** Summary of the alert symptoms for metastatic roots or spinal cord compression (MSCC) and progression of metastatic spine disease.

Neck or back nocturnal pain
Axial mechanical pain (induced or worsened by movements and under pressure relieved by lying down)
Sudden onset of axial pain
Radicular pain radiating to arms or legs associated or not with numbness, tingling, dysesthesia
Walking or balance difficulties or arms/hands weakness for impairment of one or more muscles
Bladder or bowel control disorders, urinary retention

## Data Availability

The data can be shared up on request.
